# First report of *Klebsiella quasipneumoniae* harboring *bla*_KPC-2_ in Saudi Arabia

**DOI:** 10.1186/s13756-019-0653-9

**Published:** 2019-12-19

**Authors:** Sharif Hala, Chakkiath Paul Antony, Mohammed Alshehri, Abdulhakeem O. Althaqafi, Asim Alsaedi, Areej Mufti, Mai Kaaki, Baraa T. Alhaj-Hussein, Hosam M. Zowawi, Abdulfattah Al-Amri, Arnab Pain

**Affiliations:** 1 Pathogen Genomics Laboratory, Biological and Environmental Sciences and Engineering, King Abdullah University of Science and Technology, Thuwal, Saudi Arabia; 2 Clinical Microbiology Department, King Abdullah International Medical Research Centre - Ministry of National Guard Health Affairs, Jeddah, Saudi Arabia; 3 King Saud bin Abdulaziz University for Health Sciences, Jeddah, Saudi Arabia; 4 WHO Collaborating Centre for Infection Prevention and Control, and GCC Center for Infection Control, Riyadh, Saudi Arabia; 5 UQ Centre for Clinical Research, Herston, Queensland, The University of Queensland, Queensland, Australia; 6 Global Station for Zoonosis Control, Global Institution for Collaborative Research and Education (GI-CoRE), Hokkaido University, Thuwal, Japan; 7 Red Sea Research Center, Biological and Environmental Sciences and Engineering, King Abdullah University of Science and Technology, Thuwal, Saudi Arabia

**Keywords:** MDR, *Klebsiella quasipneumoniae*, Carbapenemases, KPC-2, *bla*_KPC-2_, *Tn3*

## Abstract

**Background:**

Nosocomial infections caused by multi-drug resistant *Enterobacteriaceae* are a global public health threat that ought to be promptly identified, reported, and addressed accurately. Many carbapenem-resistant *Enterobacteriaceae*-associated genes have been identified in Saudi Arabia but not the endemic *Klebsiella pneumoniae* carbapenemases (KPCs), which are encoded by *bla*_KPC-type_ genes. KPCs are known for their exceptional spreading potential.

**Methods:**

We collected *n* = 286 multi-drug resistant (MDR) *Klebsiella spp.* isolates as part of screening for resistant patterns from a tertiary hospital in Saudi Arabia between 2014 and 2018. Antimicrobial susceptibility testing was carried out using both VITEK II and the broth microdilution of all collected isolates. Detection of resistance-conferring genes was carried out using Illumina whole-genome shotgun sequencing and PacBio SMRT sequencing protocols.

**Results:**

A Carbapenem-resistant *Enterobacteriaceae* (CRE) *Klebsiella quasipneumoniae* subsp. *similipneumoniae* strain was identified as a novel ST-3510 carrying a *bla*_KPC-2_ carbapenemase encoding gene. The isolate, designated as NGKPC-421, was obtained from shotgun Whole Genome Sequencing (WGS) surveillance of 286 MDR *Klebsiella spp*. clinical isolates. The NGKPC-421 isolate was collected from a septic patient in late 2017 and was initially misidentified as *K. pneumoniae*. The sequencing and assembly of the NGKPC-421 genome resulted in the identification of a putative ~ 39.4 kb IncX6 plasmid harboring a *bla*_KPC-2_ gene, flanked by transposable elements (IS*Kpn6-bla*_KPC-2_–IS*Kpn27*).

**Conclusion:**

This is the first identification of a KPC-2-producing CRE in the Gulf region. The impact on this finding is of major concern to the public health in Saudi Arabia, considering that it is the religious epicenter with a continuous mass influx of pilgrims from across the world. Our study strongly highlights the importance of implementing rapid sequencing-based technologies in clinical microbiology for precise taxonomic classification and monitoring of antimicrobial resistance patterns.

## Background

Beta-lactam antibiotics such as penicillins, cephalosporins, and carbapenems represent up to 60% of the available treatment options for antibiotic-resistant Gram-negative bacteria [1]. These antibiotics can be hydrolyzed by the production of extended-spectrum beta-lactamases (ESBL) and carbapenemases [2]. Carbapenem-resistant *Enterobacteriaceae* (CRE) is known to cause a variety of nosocomial infections that are associated with high mortality rates [3, 4]. Many CRE-associated genes have been identified, including the endemic *Klebsiella pneumoniae* carbapenemases (KPCs) and the New Delhi Metallo-beta-lactamases (NDMs) [3, 5]. Rapid identification and reporting of such markers could aid in minimizing the horizontal transfer of these genes to other bacterial species and ultimately preventing the spread of antibiotic resistance [6, 7]. The KPC-type enzymes, which are encoded by *bla*_KPC-type_ genes, are hard to detect by routine susceptibility screening and are known for their exceptional dissemination potential in various CREs [8]. To date, 24 variants of the *bla*_KPC-type_ genes have been reported from different countries, including the USA, Poland, South Korea, Malaysia, and Thailand [9–12]. These variants are typically identified within a *Tn3*-family of transposable elements capable of transferring resistance genes at high frequency [13].

*Klebsiella pneumoniae* was initially classified into three genetically closely-related phylogroups, and more recently, they have been classified into three distinct species: *K. pneumoniae* [KPI], *K. quasipneumoniae* [KpII], and *K. variicola* [KpIII] [14]. This distinction was possible through the comparison of their core genomes and not through conventional Multilocus Sequence Typing (MLST) and capsule genotyping [13, 14]. Due to significant overlap in their biochemical profiles, phenotypic testing using traditional clinical microbiological assays is incapable of accurately differentiating between *Klebsiella* spp. leading to false reporting of clinically isolated *K. quasipneumoniae* to be *K. pneumoniae* [13].

*K. quasipneumoniae* was initially known as a commensal intestinal colonizer. However, recent genomics-driven studies have documented it as an etiologic agent in a number of clinical *Klebsiella*-related infection cases [13, 15–17]. Clearly, such findings would underline the importance and usefulness of comparative genomics in defining the relatedness and differences between such strains, which could ultimately aid in reaching an accurate and prompt diagnosis.

Here, we report the first *bla*_KPC-2_ harboring plasmid in a *K. quasipneumoniae* strain isolated from a tertiary care hospital in the Gulf states (GCC) region, identified by a whole-genome sequencing (WGS) approach to detect multi-drug resistant (MDR) markers in a large collection of *Klebsiella spp.* isolates.

## Materials and methods

### Clinical setting and the case

As part of a larger study aiming to characterize *Klebsiella* spp*.* in a tertiary hospital in King Abdulaziz Medical City (KAMC) in Jeddah, Saudi Arabia, we collected MDR strains (*n* = 286) between 2014 and 2018. All strains were subjected to routine identification by using VITEK MS and VITEK II systems (bioMérieux Inc., Durham, NC) and were recorded in the laboratory information system as *K. pneumoniae*. Following the initial analysis of shotgun sequencing of the 286 MDR *Klebsiella* clinical isolates by Illumina sequencing technology (Illumina, USA) (data not shown), we identified a single isolate (NGKPC-421) as *K. quasipneumoniae*, which previously was falsely identified as *Klebsiella pneumoniae* by the clinical microbiology laboratory in the hospital. This isolate was obtained from a male patient in his mid-60s with chronic comorbidities admitted due to trauma-associated airway obstruction in November 2017, which promptly required a Percutaneous Tracheostomy. The patient developed nosocomial infection one-week post-surgery, and his health deteriorated gradually with symptoms mainly indicative of pneumonia. The first positive culture from the patient’s tracheostomy wound was identified as MDR *K. penumoniae* in December 2017 using VITEK II and VITEK MS. The infection persisted for a couple of months without improvement despite the antibiotic treatment and eventually caused septic shock and subsequent death. Antibiotic susceptibility testing (AST) and Minimum Inhibitory Concentration (MIC) of the NGKPC-421 isolate was conducted using the automated MICRONAUT system (Merlin Diagnostika, Germany). The AST result was interpreted using the resistance breakpoints defined by the EUCAST [18]. The resistance phenotype reported by both VITEK II and MICRONAUT methods were in complete agreement (Table 1).

**Table 1:** The AST profile and MIC of the NGKPC-421 isolate

Antimicrobial agent	MIC (mg/L)	VITEK II^a^	Micronaut^a^
Amikacin	4	S	S
Amoxicillin-clavulanate	32	R	–
Ampicillin	32	R	R
Cefazolin	30	R	R
Cefepime	4	R	R
Cefoxitin	30	–	R
Cefotaxime	2	R	R
Ceftazidime	64	R	R
Ceftriaxone	6	R	–
Chloramphenicol	4	–	S
Ciprofloxacin	0.5	R	R
Colistin	1	–	S
Fosfomycin	< 32	–	S
Gentamicin	> 16	R	–
Imipenem	4	R	R
Levofloxacin	1	–	R
Meropenem	8	R	R
Piperacillin	16	R	R
Piperacillin-tazobactam	64/4	–	R
Temocillin	< 32	–	S
Tigecycline	0.25	S	S
Trimethoprim-sulfamethoxazole	4/76	R	R

^a^VITEK II and MICRONAUT assays were repeated (*n* = 2) and (*n* = 3), respectively

R indicates resistant and S indicates susceptible to a given antibiotic based on the EUCAST guidelines [18] and (−) indicates the undetermined antimicrobial sensitivity test for a given antimicrobial agent

### Genome sequencing, assembly, and analyses

Genomic DNA of the NGKPC-421 strain was extracted from a single colony, and DNA library was prepared using the NEBNext Ultra II kit for Illumina (New England BioLabs, UK). Paired-end shotgun WGS was performed on Illumina HiSeq 4000 platform (Illumina, CA, USA). To obtain long sequence reads for assembling the complete genome, we also sequenced the high molecular-weight genomic DNA on a Pacbio RS II platform (Pacific Biosciences, CA, USA). The raw PacBio reads were first converted to fastq format by using bamtools v2.3.0 [19] and then assembled on Canu v1.6 [20] by using the default assembly settings (Additional file 1). The resulting assembly file was further polished with Pilon v1.20 [21] by supplying the sorted bam file that was generated from bwa v0.7.17 [22] mapping of Illumina reads to the initial Canu assembly (Additional file 1). The gfa output file from the Canu-assembly of NGKPC-421 was further loaded on Bandage [23] to visualize the connections between contigs so as to identify the putative chromosome and plasmid sequence-containing contigs. All contig sequences that showed no connections to the chromosome (potentially extrachromosomal in nature) were probed for the presence of signature genes that are commonly found associated with bacterial plasmids i.e., genes that encode for plasmid replication (Rep) or conjugative transfer (Tra) proteins. These identified putative plasmids that were then further analyzed on pMLST v1.4 and PlasmidFinder v2.1 web-tools [24] web-tools. Antibiotic resistance gene identification was done with the Kleborate tool v0.3.0 [25] and Resfinder [26] and confirmation of the gene annotations was made using the PATRIC online database [27].

### Phylogenetic analysis and annotation

The final assembly was used to assign core genome MLST (cgMLST) by using the *K. quasipneumoniae* BIGSdb database (http://bigsdb.pasteur.fr/Klebsiella/Klebsiella.html) [28, 29]. Available assembled complete genomes of [KPII-A], [KPII-B] *K. quasipneumoniae* reference sequences were downloaded from the NCBI database (November 2018) and aligned with identified *K. quasipneumoniae* (NGKPC-421) genome using progressiveMauve [30]. Alignment-based phylogenetic tree was constructed of *K. quasipneumoniae* subspecies (CP014154/HKUOPA4, CP034136/G747, CP034129/G4584, CP031257/L22, CP030171/A708, CP029597/ATCC 700603, CP023480/KPC-142 CP029443/CAV1947, CP029432/CAV2018, CP014156/HKUOPL4, CP014155/HKUOPJ4, CP012300/HKUOPLC, CP012252/HKUOPLA, and CP0023478/HKUOPA4) as well as outgroup strains of [KPI] *K. pneumoniae* (CP013322/CAV1193) and [KPIII] *K. variicola* (CP000964/342). Locally collinear blocks (LCBs) larger than 500 bp were selected from the full alignment file using a Perl script stripSubsetLCBs [30]. The core alignment file in .xmfa format was then converted into the .fasta format using the Perl script xmfa2fasta (https://github.com/kjolley/seq_scripts/blob/master/xmfa2fasta.pl). The core alignment (4.25 Mb) was further inspected visually for any misaligned regions. To ensure the bacterial strain type, a phylogenetic tree was generated using RAxML v8.2.3 [31] with the GTRGAMMA model, bootstrapping (1,000 replicates), and the best maximum likelihood tree inference. Additionally, a core-genome-SNP-based tree was constructed on Parsnp v1.2 from the Harvest suite [32] by using the recombination filtration (−x) and curated genome directory (−c) parameters.

## Results

**Fig. 1:**
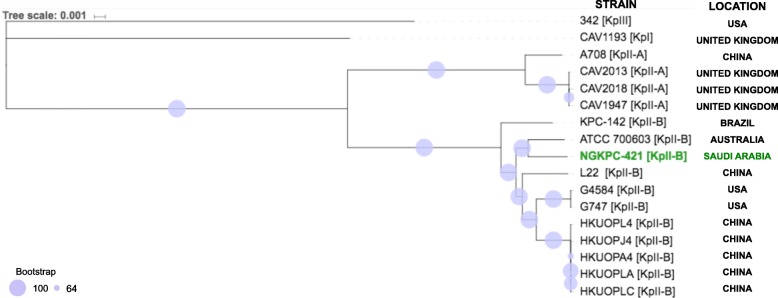
The phylogenetic tree based on segmental alignments of chromosomal sequences. The phylogenetic tree was constructed using RAxML (maximum likelihood) using reference sequences downloaded from NCBI database, including *K. quasipneumoniae* subspecies as well as strains of *K. pneumoniae* [KPI] (CAV1193) and *K. variicola* [KPIII] and viewed with the iTOL online tool v 4.4.2 [49]. Bootstrap values are represented by the size of the circles on each node. The NGKPC-421 [KPII-B] is most closely related to the ATCC strain 700,603 [KpII-B], originally isolated from Australia. *K. quasipneumoniae* subspecies (CP014154/HKUOPA4, CP034136/G747, CP034129/G4584, CP031257/L22, CP030171/A708, CP029597/ATCC 700603, CP023480/KPC-142 CP029443/CAV1947, CP029432/CAV2018, CP014156/HKUOPL4, CP014155/HKUOPJ4, CP012300/HKUOPLC, CP012252/HKUOPLA, and CP0023478/HKUOPA4) as well as outgroup strains of [KPI] *K. pneumoniae* (CP013322/CAV1193) and [KPIII] *K. variicola* (CP000964/342)

Analysis of the WGS data from the *K. pneumoniae* surveillance study identified one isolate (NGKPC-421) as *K. quasipneumoniae* subsp. *similipneumoniae* [KpII-B], that was originally misidentified as *K. pneumoniae* in the hospital laboratory*.* As shown in Fig. 1, the phylogenetic comparison of the NGKPC-421 isolate with 16 publicly available strains of *K. quasipneumoniae* 10 [KpII-B], and 4 [KpII-A], as well as *K. pneumoniae* [KpI], and *K. variicola* [KpIII], revealed a close clustering of NGKPC-421 isolate with the ATCC strain 700,603 [KpII-B], originally isolated from Australia in 2016. A separate core-genome-SNP-based tree that was constructed with Parsnp also showed identical topology (supplementary-Fig. 1). Our cgMLST analysis defined NGKPC-421 isolate as a novel *K. quasipneumoniae* subsp. *similipneumoniae* assigned as ST-3510.

Searching for genetic markers encoding antibiotic resistance showed the presence of KPC-2 gene in the NGKPC-421 isolate. The presence and location of KPC-2 gene were identified and confirmed as a result of the assembly of 6 contigs comprised of one large chromosome (~ 5.26 Mb) and four putative circular plasmids of varying sizes (Table 2). Further analysis of the complete genome also showed the presence of other antibiotic resistance genes and virulence factors (Table 2).

**Table 2:** Antibiotic resistance genes on the assembled chromosome and plasmid sequences in *K. quasipneumoniae* NGKPC-421 strain (ENA ERS3013985)

Name^a^	MLST/pMLST	Size (Kb)	CDS/GC content	Resistance genes	Predicted phenotype
Chromosome	ST3510	5255.70	*5816/57.35*	*bla*_*OKPB-3*_	β-lactam
				*oqxA*	Fluoroquinolone
				*oqxB*	Fluoroquinolone
*pKPC-421* plasmid	IncX6	39.40	*58/47.32*	*bla*_*KPC-2*_	β-lactam
*pBK30683-like* plasmid	IncFII	136.16	199/55.48	*n/a*	n/a
*pKq-NGSA-1* plasmid	IncFII(k)	204.00	303/53.31	*aac(3)lla*	Aminoglycoside
				*aac(3″)lb*	Aminoglycoside
				*aac(6′)lb-cr*	Fluoroquinolone
				*aac(6)ld*	β-lactam
				*bla*_*OXA-1*_	Phenicol
				*bla*_*CTX-M-15*_	
				*bla*_*TEM-1B*_	
				*qnrB1*	Fluoroquinolone
				*catB3 Sul2*	Sulphonamide
				*tet(A)*	Tetracycline
				*drA14*	Trimethoprim
*pK245* plasmid	IncR	134.81	*188/53.68*	*bla*_*CTX-M-14b*_	β-lactam

^a^Assembly of *K. quasipneumoniae* NGKPC-421 sequences resulted in 6 contigs comprising of 1 chromosome and 4 putative circular plasmids (Note: 2 contigs represent the *pKq-NGSA-1* plasmid)

**Fig. 2:**
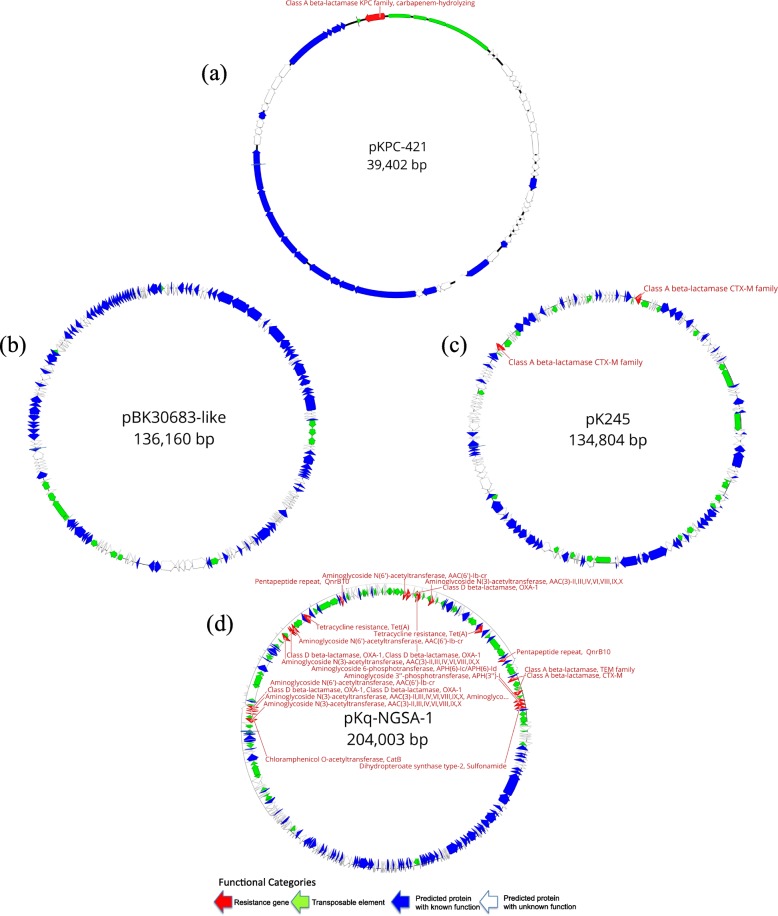
Schematic gene organization of all 4 putative circular plasmids in NGKPC-421. **a** pKPC-421 plasmid, **b** *pKq-NGSA-1* plasmid, **c** *pK245* plasmid, and **d** *pBK30683* plasmid

**Fig. 3:**
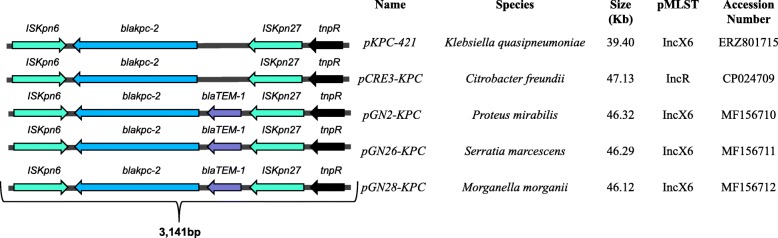
Schematic of gene arrangements of the *bla*_KPC-2_ flanked by *Tn3*-based transposon in plasmids across different species. *IS481* and *IS1182* family of genes harboring transposase and resolvase genes and insertion sequences, i.e., IS*Kpn6*, and IS*Kpn27* in 5 plasmids compared to the pKPC-421 of *K. quasipneumoniae*. This fragment has been previously identified in various plasmids in several Gram-negative species from China. The cassette of S*Kpn6-bla*_KPC-2_-IS*Kpn27* can be found in similar IncX6 plasmids (included in Fig. 2) as well as multiple types of plasmids across various bacterial species

Four putative plasmids identified in the NGKPC-421 isolate were annotated as *pKPC-421, pBK30683-like, pK245*, and a potentially novel plasmid *pKq-NGSA-1* (Fig. 2). The plasmid *pKPC*-421 (~ 39.4 Kb) belongs to the Inc6X group, where the *bla*_KPC-2_ gene is flanked by genes belonging to the *Tn3*-based transposon family (*ISKpn6 and ISKpn27)* (Fig. 3). Notably, a public database search of the *ISKpn6*-*bla*_KPC-2_-*ISKpn27* harboring sequences showed its presence on several plasmids across numerous bacterial species previously reported from many countries but not from the Gulf region (Fig. 3). The largest plasmid *pKq-NGSA-1* (~ 204 Kb), represented by two contigs, was found to be an *IncFII*(k), harboring the majority of resistance genes, including *bla*_OXA-1_, *bla*_CTX-M-15_, and *bla*_TEM-1B_ in multiple copies (Table 2). The second-largest plasmid *pBK30683-like* (~ 136 Kb), found as a single contig, had no known antibiotic resistance genes present. The fourth plasmid *pK245* (~ 135 Kb) belonged to the *IncR* group and was found to harbor *bla*_CTX-M-14b_ (Table 2).

## Discussion

Although *K. quasipneumoniae* was initially considered as a benign human gut commensal bacteria, recent reports suggest its involvement in potentially fatal infections [15, 16, 33]. Despite being rarely documented, *K. quasipneumoniae* appears to be capable of undergoing homologous recombination with other *Klebsiella* spp., allowing it to exchange both chromosomal and plasmid-borne antibiotic resistance genes [14]. Additionally, *KPC-positive* isolates co-producing Metallo-β-lactamase enzymes are known to be associated with diverse mobile genetic elements causing large outbreaks in China, Europe and the USA [34–37]. Our data demonstrate that *bla*_KPC-2_, in addition to other carbapenemase resistance genes, is present in the Gulf region and that *K. quasipneumoniae subsp. similipneumoniae* could be misidentified as *K. pneumoniae* in clinical laboratories [38].

This finding should encourage further surveillance within Saudi Arabia and neighboring countries for additional occurrences of such resistance markers. This is particularly of epidemiological significance because the *bla*_KPC-2_ was found in an IncX6 plasmid (*pKPC*-421) that is known to circulate among *Enterobacteriaceae* species [39]. The *bla*_KPC-2_ was found to be flanked by transposons, which could have major implications on the spread of antibiotic resistance within the region. Furthermore, the isolate carried other plasmids (*pKq-NGSA-1, pBK30683*, and *pK245*) containing various other genes that encode for antibiotic resistance. While such genes are known to be present in clinical CRE-associated *K. pneumoniae* strains [5, 40, 41], none of the plasmids included here were reported nor genetically characterized prior to this study in the Gulf region [42–45].

Although the presence of various β-lactamase genes among *Enterobacteriaceae* such as *bla*_SHV-1_, *bla*_TEM-1,_ *bla*_CTX-M-1_, *bla*_NDM-1_ and *bla*_OXA-48_ in Saudi Arabia and the Gulf region has been previously reported [42–45], though this is the first report of a KPC-type producing *K. quasipneumoniae*. The discovery of a misidentified MDR *K. quasipneumoniae* isolate is worrisome, suggesting the need to update identification methods and the consideration of the implementation of rigorous molecular surveillance [46]. The data reported in this study support the need to investigate the emergence and spread of KPC-producing strains from the region. Furthermore, the inability of conventional laboratory techniques to detect KPC-2 gene in this CRE isolate increases the need for routine reconnaissance using WGS. Considering the importance of Saudi Arabia as a host of one of the largest recurring mass gathering events (*i.e*. Hajj and Umra pilgrimage) with 7 million pilgrims from more than 180 countries [47], infection control efforts are essential so as to monitor the potential spread of such KPC-producing *Enterobacteriaceae*. Current conventional molecular microbiology laboratory diagnostic methods are sufficient for diagnosing infectious diseases, and WGS may be used as an additional confirmatory tool [48]. Although performing WGS techniques would essentially require access to expensive equipment and reagents in addition to computational infrastructures and relevant expertise; our study re-emphasizes that the introduction of clinical genomics-driven surveillance methods in the GCC region could aid in accurate surveillance and confirmed diagnoses of etiologic agents and the determination of their antibiotic resistance markers. This is of particular public health relevance for monitoring the potential spread of KPC-producing *Enterobacteriaceae* during mass gathering events such as Hajj and Umrah in Saudi Arabia.

## Supplementary information

**Additional file 1:** Command-lines for the tools

## Data Availability

The raw reads and/or assembly files from this study are publicly available at the European Nucleotide Archive (ENA) under the study accession number PRJEB30599 http://www.ebi.ac.uk/ena/data/view/PRJEB30599 and at the Zenodo open-access repository- 10.5281/zenodo.2583210

## Abbreviations

CRE: Carbapenem Resistant *Enterobacteriaceae*
ESBL: Extended-spectrum beta-lactamase
KPC: *Klebsiella pneumonia* Carbapenemase
MIC: Minimum Inhibitory Concentration
WGS: Whole Genome Shotgun Sequencing
