# Supplementary material for: First report of Klebsiella quasipneumoniae harboring blaKPC-2 in Saudi Arabia
Source: Antimicrob Resist Infect Control. 2019 Dec 19;8:203. doi: 10.1186/s13756-019-0653-9 (PMC6923860; doi:10.1186/s13756-019-0653-9)
Supplement: Supplementary file 1 — Additional file 1: Command-lines for the tools [file 13756_2019_653_MOESM1_ESM.docx]

**Command-lines for the tools used:**

**Canu v1.6 assembly and polishing with Pilon v1.20:**

canu -p NGKPC_421 -d /directory genomeSize=5.89M -pacbio-raw NGKPC_421,subreads.fastq useGrid=false gnuplotTested=true)

java -jar pilon-1.20.jar --genome NGKPC421.contigs.fasta –frags Illumina_421_contigs.sorted.bam --output Pilon_421

**Core-genome-SNP tree construction on Parsnp v1.2**

parsnp -r genomes_dir/NGKPC_421.fasta -d genomes_dir -p 8 -o KP_Parsnp_output -x -c)
